# Novice Female Exercisers Exhibited Different Biomechanical Loading Profiles during Full-Squat and Half-Squat Practice

**DOI:** 10.3390/biology10111184

**Published:** 2021-11-15

**Authors:** Xin Li, Ntwali Adrien, Julien S. Baker, Qichang Mei, Yaodong Gu

**Affiliations:** 1 Faculty of Sports Science, Ningbo University, Ningbo 315211, China; 2011042028@nbu.edu.cn (X.L.); 2014040001@nbu.edu.cn (N.A.); 2 Research Academy of Grand Health, Ningbo University, Ningbo 315211, China; 3 Centre for Health and Exercise Science Research, Department of Sport and Physical Education, Hong Kong Baptist University, Hong Kong 999077, China; 4 Auckland Bioengineering Institute, The University of Auckland, Auckland 1010, New Zealand; 5 Faculty of Engineering, University of Szeged, 6720 Szeged, Hungary

**Keywords:** squat, half-squat, female exerciser, OpenSim, biomechanics, SPM1D

## Abstract

**Simple Summary:**

This study adapted a customized OpenSim model aiming to analyze the loadings difference between full-squat and half-squat in novice females. The joint moment and joint angle of the hip, knee, and ankle increase significantly in the full-squat, which might increase the risks of potential injury. In the case of training, the cohort of young females could perform half-squat practice when muscle strength is insufficient. This study may present implications for the design of novice strength training programs and the formulation of rehabilitation plans.

**Abstract:**

Background: Females with different practice experience may show different body postures and movement patterns while squatting in different depths, which may lead to changes of biomechanical loadings and increase the risks of injuries. Methods: Sixteen novice female participants without squat training experience participated in this study. A 3D motion capture system was used to collect the marker trajectory and ground reaction force data during bodyweight squatting in different depths. The participants’ kinematic data and joint moment were calculated using OpenSim’s inverse kinematics and inverse dynamics algorithm. In this study, authors adapted a model especially developed for squatting and customized the knee joint with extra Degree-of-Freedom (DoF) in the coronal and horizontal plane with adduction/abduction and internal/external rotation. A paired-sample t-test was used to analyze the difference of joint range of motions (ROM) and peak moments between full-squat (F-SQ) and half-squat (H-SQ). One-Dimensional Statistical Parametric Mapping (SPM1D) is used to analyze the difference of joint angle and moment between the process of squatting F-SQ and H-SQ. Results: (1) Compared with H-SQ, F-SQ showed larger ROM in sagittal, coronal, and transverse planes (*p* < 0.05). (2) SPM1D found that the difference in joint angles and joint moments between F-SQ and H-SQ was mainly concentrated in the mid-stance during squatting, which suggested the difference is greatly pronounced during deeper squat. (3) Peak hip extension moment, knee extension moment, hip adduction moment, and plantar flexion moment of F-SQ were significantly higher than H-SQ (*p* < 0.05). (4) Difference of hip and knee extension moments and rotation moments between the F-SQ and H-SQ were exhibited during descending and ascending. Conclusions: The study found that novice women had larger range of joint motion during the F-SQ than H-SQ group, and knee valgus was observed during squatting to the deepest point. Greater joint moment was found during F-SQ and reached a peak during ascending after squatting to the deepest point. Novice women may have better movement control during H-SQ. The findings may provide implications for the selection of lower limb strength training programs, assist the scientific development of training movements, and provide reference for squat movement correction, thus reducing the risk of injury for novice women in squatting practice.

## 1. Introduction

The squat is one of the closed kinetic chain exercises [1,2,3]. The squat process involves more than 200 muscles and demands multi-joint coordination [2,4]. The squat is widely conducted during resistance training, which could increase lower limb strength, prevent sports injuries, and improve sports performance [5,6,7,8,9]. It is also used in rehabilitation therapy to assess physical flexibility and symmetry [6] and postoperative rehabilitation training [1,7]. In daily life, the squat is also a component of physical activity [10,11].

Practice of squats in a correct manner will not cause injuries [2], but the incorrect squatting technique and overload will increase the risk of injuries [4,12]. Bodyweight and barbell squats are common methods used during squatting practice [5,7]. Fifty to eighty-five percent of one repetition max (RM) barbell squats is usually performed for strength training [13]. Compared with the barbell deep squat, the bodyweight squat has lower load, which is relatively safe [14]. The bodyweight squat is generally used in novice and rehabilitation training [10]. The movement patterns are differences between males and females during squat training [15]. Females have different muscle activation and neuromuscular control patterns during squatting [16,17] Kinematic differences between males and females are one of the high-risk factors for lower limb injuries among female squat practicers [15]. 

Research has revealed that different depths of squats may result in different joint kinematics, dynamics, and muscle activities [6,9]. Full squats (F-SQ) may enhance flexibility and improve athletic performance [12]. Squats are usually performed at a shallow depth (knee flexion 0–60°) during rehabilitation training, because the injury risk to the soft tissue in the knee joint may increase during high flexion [5,9,18]. In addition, the increase of squat depth will lead to the rise in the moment of the hip joint, knee joint, and ankle joint [12,19,20], which may lead to related sports injuries. However, few current studies have contrary findings [21,22,23], for example, Flores et al. [21] showed that the peak knee extensor moment (pKEM) lack difference between 90, 110, and 135° of knee flexion in the bodyweight demonstrated. These participants were trained females, as it is possible that differences in squatting mechanics would be evident between trained and untrained populations [23]. At present, there is still no consensus on how much depth should be employed in strength training of novice females and rehabilitation of patients with knee joint injuries [23].

In addition, one limitation to most squatting studies that quantified joint and segmental angles and joint moments is that a two-dimensional (2D) analysis was employed to record the sagittal movements. 2D motion capture may not be suitable for performance assessment of any motion that is not purely uniplanar, such as the knee valgus motion at the knee. This motion, in reality, is a movement not only comprising of knee abduction and hip adduction in the frontal plane but also hip internal rotation and tibia external rotation in the transverse plane [24] This study updated a customized OpenSim squat model [25,26] with extra frontal and horizontal motions, analyzing the three dimensions (3D) motion of the knee joint and hip joint [26,27,28].

The knee flexion in the sagittal plane was employed to define squat depth [2,9]. Therefore, this study was aimed to investigate biomechanical difference of squatting depths in novice squatting practice females. The two different squat styles include F-SQ: deep squat with the maximum depth while keeping the neutral position of the spine [2], and half-squat (H-SQ): knee angle reaches 90° [9]. It was hypothesized that F-SQ may produce greater lower limb peak joint moments than the H-SQ group.

## 2. Materials and Methods

### 2.1. Participants

Sixteen female novice females were recruited for this study, including age: 22 ± 2.1 years, weight: 61.4 ± 3.2 kg, height: 1.63 ± 0.06 m, and BMI: 25.32 ± 3.27 kg/$m^{2}$. Limb dominance was confirmed via preference of ball kicking using both limbs, and the preferred limb was defined as the dominant limb. There were no lower limb diseases or injuries in the six months prior to the test. The participants did not eat for 2 h before the experiment, and any type of ingestion of caffeine and alcohol was forbidden within 24 h. All participants understood the purpose and significance of the research and signed an informed consent form. The Ethics Committee from the research institute in Ningbo University approved this test.

### 2.2. Instruments

**Figure 1:**
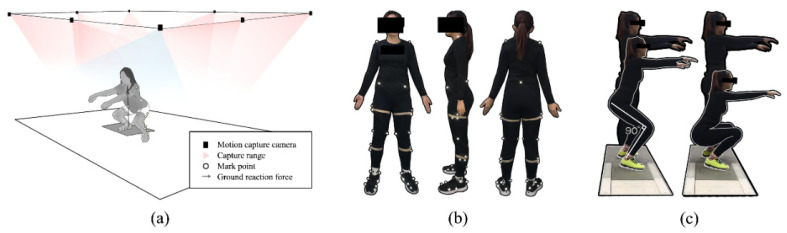
Experimental process, (**a**) Participant motion capture setup, (**b**) Illustration of each maker placement on the front, the right, the left, and the rear of the participants, (**c**) F-SQ and H-SQ practice.

An eight-camera Vicon motion capture system (Vicon Metrics Ltd., Oxford, UK) was used to capture the motion trajectory. The embedded AMTI force measuring board (AMTI, Watertown, MA, USA) was used to record the ground reaction forces synchronously, with frequencies of 200 Hz and 1000 Hz respectively, as shown in Figure 1a. The 37-reflective marker-set established previously, were used to track F-SQ and H-SQ processes [29,30], as shown in Figure 1b.

### 2.3. Experimental Protocol

Before the squat test, the participants were instructed to follow their normal warm up routine for 10 min. The F-SQ and H-SQ squatting tests were performed randomly. In the process of F-SQ and H-SQ, the dominant limb of all participants stepped on the force platform, as shown in Figure 1c.

Previous studies showed that station distance, knee joint alignment, foot position, and lumbar position could affect the lower limb biomechanics during squatting [1,31], so the influencing factors were controlled in the process of squatting. All participants were verbally instructed with a demonstration, and practice trial were performed to ensure the completion of bodyweight squat. The distance between the feet were twice the distance between the anterior superior iliac spine [32], and the knee should track over the toes throughout the squat motion without knee displacement either medially or laterally. The upper body was kept vertical to the ground during squatting, and the feet were not allowed to leave the ground during squatting. Before the start of H-SQ, the participants crouched slowly and controllably until the protractor showed that the knee angle reached 90, and an elastic belt was then set at this height to obtain a required squat depth. The squatting stance was further divided into two phases [16], the descending phases from upright to the deepest position (0~50%), and the ascending periods the from the deepest to upright position (51~100%). In the test of H-SQ, after squatting from an upright posture, the participants received feedback from the elastic belt [20]. They then changed from descending to ascending phases until recovering in an upright posture.

In the test of F-SQ, after hearing the start command, the participants started the squat and made the deepest squat until the deepest position of the pelvis was obtained. The participants then resumed the standing posture. Each action was used to collect three successful data sets for analysis. The participants had a 10-min rest between two different depth tests to ensure complete recovery.

### 2.4. Outcome Measures

Joint angles and moments included hip flexion/extension, adduction/abduction, internal/external rotation, knee flexion/extension, adduction/abduction, internal/external rotation, and ankle dorsiflex/plantar flexion. The ROM in the hip, knee, and ankle were computed to illustrate joint flexibility. The flexion of the sagittal plane, the adduction on the coronal plane, and the internal rotation in the sagittal were defined as positive.

### 2.5. Data Processing and Statistical Analysis

According to Winter’s [33] description of the selected frequency for filtering biomechanical signals, the residual analysis of data were carried out in subsets to determine the most appropriate signal-to-noise ratio. Marker trajectories and ground reaction forces were filtered by zero-delay fourth-order Butterworth low-pass filter at 12 Hz and 30 Hz, respectively. The data were converted into a recognizable format in OpenSim 4.2v by Matlab and then imported into OpenSim for data post-processing. OpenSim is an open-source platform for generating and executing dynamic simulation and analysis, which provides tools for solving inverse kinematics and inverse dynamics [27,28,34]. The model was adjusted based on the open-source squat model to prevent the muscles from passing through the bones and allow higher joint mobility (ROM) [13,18] The muscle-tendon slake employed Hill’s muscle model and followed the relationships between muscle force and strain. OpenSim can match marker trajectories collected in the motion capture system to the virtual makers in the model, enabling authentic and reliable musculoskeletal model [13,31]. OpenSim has been widely used in squatting studies, such as comparing lower limb movement and load differences between Asians and Caucasians [26], designing astronaut exercise programs in weightlessness [35], comparing neuromuscular activity between two squatting motions [36], and analyzing cruciate ligament and selected muscle loads [27]. The model was scaled using each subject’s marker point position and weight in static calibration. The starting and ending points and moment arms of the muscles of the universal model were matched with the participants. In accordance to the root mean square (RMS) error value (less than 0.02) between the experimental mark and the virtual mark in the model, the static weight of each mark was manually adjusted. The joint angles of the F-SQ and H-SQ were calculated using the inverse kinematics (IK) calculation tool in OpenSim, and the least square method was used to optimize the results and minimize the error between the experimental markers and the virtual markers. The Inverse dynamics (ID) algorithm in OpenSim was used to calculate the net moment of the hip, knee, and ankle joint. The weight of the subject was used to standardize the joint moment. The unit of the joint moment was (Nm/kg).

One-Dimensional Statistical Parametric Mapping (SPM1D) has become considerably more prevalent and practicable. Unlike the traditional statistical methods that analyze the differences in peak value, this method can base the continuous data analyzed differences of the squat motion [26,37]. To date, there have yet to be many biomechanical investigations that have examined the difference of varying depth in kinetics and kinematics during the squat using an SPM1D.

The squat process can be divided into two stages [21], descending and ascending. The descending stage can be defined as moving from the upright state descending to the deepest position (0~50%), and the ascending stage can be defined as restoring the upright state from the deepest position (51~100%) [10]. Data processing and statistical analysis were processed in Matlab 2018a (Mathworks, Natick, MA, USA). SPM1D is a continuous data analysis method, due to the one-dimensional (1D) time-varying characteristics of joint moments. The cubic spline interpolation method was used to interpolate the whole cycle from a squat posture into a data set with 101 data points and then the average of the three trials from each action was obtained [37]. An independent sample test package in SPM1D was used for statistical analysis and the significance level was set at *p* < 0.05.

## 3. Results

### 3.1. Kinematics

As shown in Table 1, ROMs were significantly larger (*p* < 0.001) during F-SQ than H-SQ for the lower limb joint.

**Figure 2:**
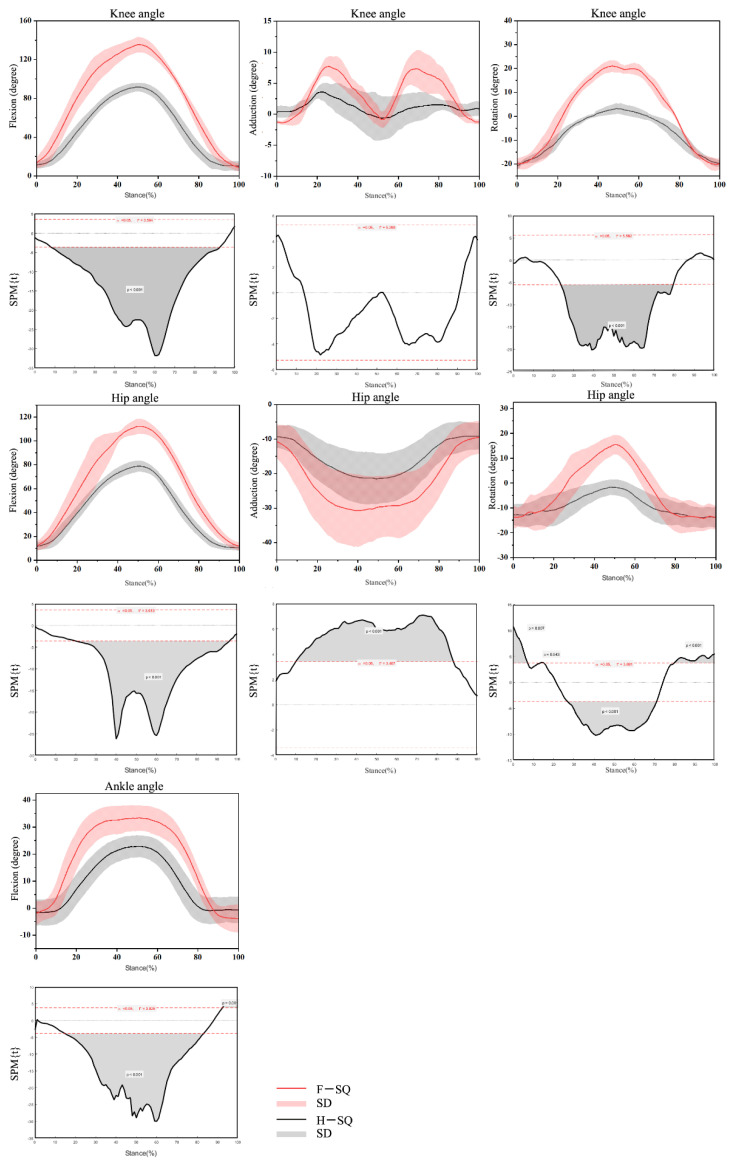
Waveform of angles in the hip, knee and ankle joints during F-SQ and H-SQ with highlighted statistics using SPM1D.

Lower extremity joint kinematic patterns of the hip, knee, and ankle joints were plotted against the squat percentage for the F-SQ/H-SQ conditions (Figure 2). In terms of the comparison of time-varying joint angles during the squat, differences were found in the knee joint that F-SQ exhibited larger knee joint flexion angle (*p* < 0.01, 8.68–93.04%) and knee joint internal rotation angle (*p* < 0.001, 24.35–79.76%).

In terms of the comparison of time-varying hip angles during the squat, differences were found in the hip flexion that F-SQ exhibited larger flexion (*p* < 0.001, 21.88–95.70%) and internal rotation (*p* < 0.001, 27.19–71.08%) and hip abduction (*p* < 0.001, 10.34–88.62%) joint angle than H-SQ.

The statistical results of SPM1D showed F-SQ had a larger ankle dorsiflexion angle during 15.07–83.02% squat phases (*p* < 0.001).

### 3.2. Kinetics Analysis

**Figure 3:**
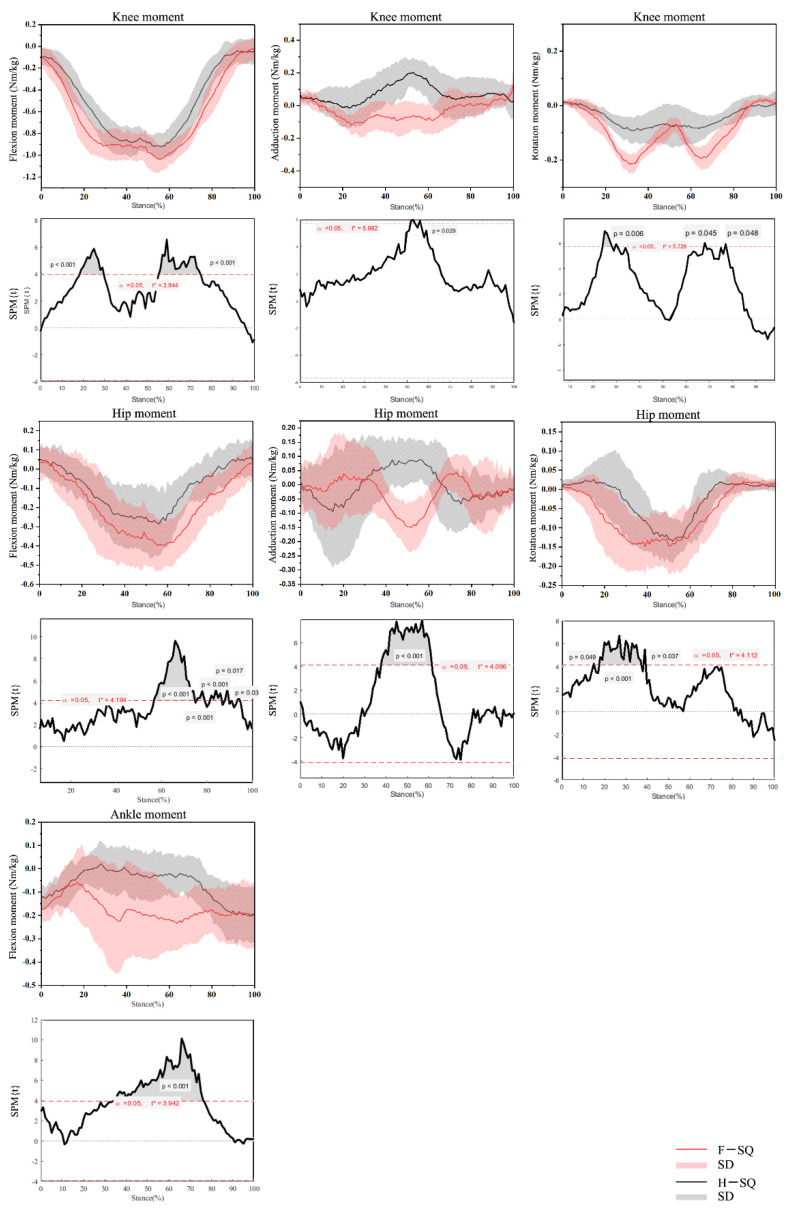
Waveform of moments in the hip, knee and ankle joints during F-SQ and H-SQ with highlighted statistics using SPM1D.

As shown in Figure 3, the F-SQ knee joint extension moment was more considerable than H-SQ and was significant between 18.22–29.76% and 54.90–74.98% of the whole action cycle (*p* < 0.001). The H-SQ knee joint abduction moment was bigger than F-SQ and was significant between 51.6–56.79% of the whole action cycle (*p* < 0.001). The F-SQ knee joint external rotation moment was more considerable than H-SQ and was significant between 17.96–25.62% of the whole action cycle (*p* < 0.001).

Differences in the joint moments showed that F-SQ had greater hip extension moment during 56.63–94.72% (*p* < 0.001) of the squatting stance. The F-SQ hip joint abduction moment was more considerable than H-SQ and was significant between (37.87–61.43%) of the whole action cycle (*p* < 0.001). Significantly greater hip external rotation moments were also found in F-SQ during 17.45–61.43% (*p* < 0.001) of the squatting stance.

The statistical results of SPM1D show H-SQ exhibited larger ankle plantarflexion moment during 34.03–76.44% squat phases (*p* = 0.004).

## 4. Discussion

This study aimed to compare and comprehensively analyze the differences of lower limb biomechanics in the sagittal plane, coronal plane, and horizontal plane of novice women during the H-SQ and F-SQ squat practice using the OpenSim platform with a customized musculoskeletal model. F-SQ and H-SQ are often used to strengthen lower limbs and as method for rehabilitation training [1,5,6,7,8,9]. The joint kinematics, kinetics, and muscle activity may be different at different depths of squat [6,7,8,9]. With the increase of squatting depth, the moment of hip, knee, and ankle also increased, thus increasing the probability of injury [12,19,20]. In contrast to the results of previous studies, in the latest study by Flores et al. [21], with 19 female participants that with the increase of squat depth, the peak moment of the knee joint did not increase significantly. However, it should be noted that all participants were experienced squat practicer. According to Salem et al.’s report [23], there are obvious differences in squat mechanism between novice people with experience women, so the difference in research results may be related to the training experience. [21,23], but the studies on novice women are still scarce, and previous studies are mostly focused on a single plane, either the sagittal plane or coronal plane, while the comprehensive analysis of three plane movements are still very few, further research is needed.

### 4.1. Kinematics

The results showed that the hip, knee, and ankle joints of the participants showed a larger ROM during F-SQ than that of H-SQ. Mei et al. reported that the change of ROM during squatting affects the development of strength, the speed of strength development, the activation and synchronization of motor units, and the stability of dynamic joints [26]. Therefore, it is necessary to make a comparative analysis of the kinematic differences between F-SQ and H-SQ in novice women.

In the F-SQ, the ROM of the ankle on the sagittal plane was significantly larger than that of the H-SQ, which indicated that F-SQ had higher requirements for the dorsiflexion ability of the ankle. In the studies of Agarwal et al. [11] and Rhea et al. [38], they indicated that with the increase of squatting depth, when the movement ability of the ankle joint was not enough to maintain the contact between the foot and the ground, the torso showed a more forward posture to maintain the stability of the center of gravity. This compensation mechanism may lead to an increase in the sheer force received by the lumbar vertebrae, thus increasing the risk of injury [39,40]. In addition, it is worth noting that in F-SQ, when the flexion angle of the knee joint reached the maximum, i.e., squatting to the deepest, the femur and tibia was in a state of internal rotation, the knee joint showed a slight abduction angle, the hip joint showed a transient trend of adduction, which meant that when squatting to the deepest point, the subject’s knee joint showed valgus, which was a common postural error. It may increase the pressure on the anterior cruciate ligament and increase the risk of knee injury [41,42]. However, this phenomenon was not observed during H-SQ, which may be because the technical maturity and the neural mechanism of controlling movements of the novice are different from those of those with training experience when performing F-SQ movements with a larger range of movements because novices seem to prefer to lift with high acceleration while neglecting the control of movements [43,44]. However, this phenomenon cannot be further analyzed by existing evidence, as it is not the purpose of this study and will be further investigated in the future. Therefore, we believe that there are significant differences in lower limb kinematic parameters between novice women during F-SQ and H-SQ, and knee valgus and excessive forward tilt of the torso may occur during F-SQ, especially when squatting to the deepest place, which may lead to a higher risk of injury.

### 4.2. Kinetics

The results of this study showed that compared with H-SQ, participants had greater extension moment of hip, knee, and ankle, greater abduction moment of hip and knee, and external rotation moment of knee and hip during F-SQ. The excessive joint moment may lead to injury. However, in the published studies, the studies on the dynamic parameters of novice female squatting are mostly focused on the comparison of a peak moment. Therefore, it is necessary to compare and analyze the changes of moment in the whole action cycle.

Previous studies showed that there was a direct linear relationship between knee joint peak moment and patellofemoral joint reaction force. As excessive patellofemoral joint reaction force was related to a variety of knee joint diseases such as patellofemoral pain syndrome, articular cartilage degeneration, and chronic knee joint pain, therefore, excessive knee joint moment may increase the risk of joint injury. In contrast to the results observed in this study, Flores et al. [21] and Golfeshan et al. [36] showed that there was no statistically significant difference in knee joint peak moment among squat exercises at different depths without extra load, which may be due to the training experience. The participants included in this study are novices with no lower limb strength training experience, while in the study of Flores et al. and Golfeshan et al., the participants are all women with some training experience, and there is ample evidence that participants with strength training experience usually have stronger muscle strength [45,46] and better movement control ability [5,47]. In addition, the study of Wallace et al. [12] showed that the knee extension moment increased with the increase of knee flexion angle during squatting. However, in this study, the extension moment of the hip joint and knee joint appeared between 50% and 60% of the action cycle, i.e., the moment of lifting after squatting to the deepest point, and the extension moment of hip and ankle joint and the abduction moment of hip joint and knee joint of F-SQ were significantly higher than that of H-SQ at the moment of getting up. This may be because novice women in the squat lift moment there will be a greater instantaneous acceleration to complete the action and reduce the sense of control of the action [44], and when F-SQ to the deepest, the thigh and calf contact may produce a moment in the same direction as the quadriceps femoris, thus reducing the knee extension moment of the knee [5]. Therefore, compared with F-SQ, novice women have significantly smaller joint torque during H-SQ, which may indicate that novice women have better control of movement and a lower risk of injury during H-SQ.

### 4.3. Limitations

There are a few limitations that should be considered in the current study. (1) The participants were young females. If these results are extended to all female populations, further work is needed to confirm the findings outlined here and may need to investigate biomechanical differences across different age-group. (2) The study results mainly investigate the ROM and moment of the joint, and there is no study on the degree of muscle activation and muscle strength. This paper was a pilot study, and further research will be needed to investigate the findings further. Although we do not know what kind of moment will harm the knee joint, the findings of this study could help the design and implementation of squat training.

## 5. Conclusions

The following results were obtained using biomechanical analysis of the lower limbs of novice females performing the H-SQ and F-SQ: (1) Compared with H-SQ, the participants had a greater ROM during F-SQ and showed knee valgus when squatting to the deepest place, indicating that novice women had weaker control over F-SQ than H-SQ. (2) During F-SQ, novice women showed that the extension moment of the hip, knee, and ankle joint, and the abduction moment of the hip joint and knee joint were significantly larger than those of H-SQ, which may increase the potential risk of injury. (3) At the moment when the participants squatted to the deepest position, the extension moment of hip and ankle joint and the abduction moment of hip joint and knee joint reached the peak and were significantly greater than H-SQ. These findings indicated that novice women tend to accelerate the completion of movements at the deepest position, and this phenomenon may weaken the consciousness of movement control and lead to movement deformation and injury.

## Figures and Tables

**Table 1:** Range of joint motion.

		H-SQ	F-SQ	Mean Difference (95%CI)	F	t	*p*-Value
Knee	sagittal	82.64 ± 8.40	126.93 ± 7.50	−44.29 (−50.06, −38.53)	0.38	−15.69	*p* < 0.001
	coronal	6.52 ± 1.16	10.42 ± 2.67	−3.23 (−6.15, −3.12)	0.76	−4.86	*p* < 0.001
	horizontal	24.68 ± 4.34	42.02 ± 16.50	−17.34 (−21.49, −13.19)	4.19	0.07	*p* < 0.001
Hip	sagittal	69.47 ± 7.15	102.55 ± 7.37	−33.07 (−38.40, −27.75)	0.47	−12.68	*p* < 0.001
	coronal	13.63 ± 4.52	22.85 ± 7.68	−9.22 (−13.77, −4.67)	6.79	−4.14	*p* < 0.001
	horizontal	13.88 ± 3.32	38.47 ± 11.77	−24.60 (−30.84, −18.35)	16.11	−8.05	*p* < 0.001
Ankle	sagittal	25.78 ± 3.89	38.73 ± 2.96	−12.95 (−15.45, −10.46)	0.80	−10.60	*p* < 0.001

## Data Availability

The data that support the findings of this study are available on reasonable request from the corresponding author. The date is not publicly available due to privacy or ethical restrictions.

## References

[1] Khaiyat O.A., Norris J. (2018). Electromyographic activity of selected trunk, core, and thigh muscles in commonly used exercises for ACL rehabilitation. J. Phys. Ther. Sci..

[2] Martínez-Cava A., Morán-Navarro R., Sánchez-Medina L., González-Badillo J.J., Pallarés J.G. (2019). Velocity-and power-load relationships in the half, parallel and full back squat. J. Sports Sci..

[3] Escamilla R.F., Fleisig G.S., Zheng N., Barrentine S.W., Wilk K.E., Andrews J.R. (1998). Biomechanics of the knee during closed kinetic chain and open kinetic chain exercises. Med. Sci. Sports Exerc..

[4] O’Reilly M.A., Whelan D.F., Ward T.E., Delahunt E., Caulfield B.M. (2017). Technology in Strength and Conditioning: Assessing Bodyweight Squat Technique with Wearable Sensors. J. Strength Cond. Res..

[5] Escamilla R.F. (2001). Knee biomechanics of the dynamic squat exercise. Med. Sci. Sports Exerc..

[6] Cotter J.A., Chaudhari A.M., Jamison S.T., Devor S.T. (2013). Knee joint kinetics in relation to commonly prescribed squat loads and depths. J. Strength Cond. Res. Natl. Strength Cond. Assoc..

[7] Schoenfeld B.J. (2010). Squatting Kinematics and Kinetics and Their Application to Exercise Performance. J. Strength Cond. Res..

[8] Kellis E., Arambatzi F., Papadopoulos C. (2005). Effects of load on ground reaction force and lower limb kinematics during concentric squats. J. Sports Sci..

[9] Hartmann H., Wirth K., Klusemann M. (2013). Analysis of the Load on the Knee Joint and Vertebral Column with Changes in Squatting Depth and Weight Load. Sports Med..

[10] Li G., Most E., DeFrate L., Suggs J., Gill T., Rubash H. (2004). Effect of the posterior cruciate ligament on posterior stability of the knee in high flexion. J. Biomech..

[11] Agarwal B., Deursen R., Mullerpatan R. (2018). Influence of habitual deep squatting on kinematics of lower extremity, pelvis and trunk. Int. J. Health Rehabil. Sci..

[12] Wallace D.A., Salem G.J., Salinas R., Powers C.M. (2002). Patellofemoral Joint Kinetics While Squatting with and without an External Load. J. Orthop. Sports Phys. Ther..

[13] Santos Catelli D. (2019). Femoroacetabular Impingement Syndrome and Total Hip Arthroplasty: Joint Biomechanics before and after Surgery.

[14] Thiebaud R.S., Funk M.D., Abe T. (2014). Home-based resistance training for older adults: A systematic review. Geriatr. Gerontol. Int..

[15] Zawadka M., Smolka J., Skublewska-Paszkowska M., Lukasik E., Bys A., Zielinski G., Gawda P. (2020). Sex-dependent differences in single-leg squat kinematics and their relationship to squat depth in physically active individuals. Sci. Rep..

[16] Lephart S.M., Ferris C.M., Riemann B.L., Myers J.B., Fu F.H. (2002). Gender Differences in Strength and Lower Extremity Kinematics During Landing. Clin. Orthop. Relat. Res..

[17] Mehls K., Grubbs B., Jin Y., Coons J. (2020). Electromyography Comparison of Sex Differences During the Back Squat. J. Strength Cond. Res..

[18] Escamilla R.F., Fleisig G.S., Zheng N., Lander J.E., Barrentine S.W., Andrews J.R., Bergemann B.W., Moorman III C.T. (2001). Effects of technique variations on knee biomechanics during the squat and leg press. Med. Sci. Sports Exerc..

[19] Damsgaard M., Rasmussen J., Christensen S.T., Surma E., de Zee M. (2006). Analysis of musculoskeletal systems in the AnyBody Modeling System. Simul. Model. Pr. Theory.

[20] McBride J.M., Kirby T.J., Haines T.L., Skinner J. (2010). Relationship between Relative Net Vertical Impulse and Jump Height in Jump Squats Performed to Various Squat Depths and With Various Loads. Int. J. Sports Physiol. Perform..

[21] Flores V., Becker J., Burkhardt E., Cotter J. (2020). Knee Kinetics During Squats of Varying Loads and Depths in Recreationally Trained Women. J. Strength Cond. Res..

[22] Bryanton M.A., Kennedy M.D., Carey J.P., Chiu L.Z. (2012). Effect of Squat Depth and Barbell Load on Relative Muscular Effort in Squatting. J. Strength Cond. Res..

[23] Salem G.J., Powers C.M. (2001). Patellofemoral joint kinetics during squatting in collegiate women athletes. Clin. Biomech..

[24] Shirey M., Hurlbutt M., Johansen N., King G.W., Wilkinson S.G., Hoover D.L. (2012). The influence of core musculature engagement on hip and knee kinematics in women during a single leg squat. Int. J. Sports Phys. Ther..

[25] Catelli D.S., Wesseling M., Jonkers I., Lamontagne M. (2018). A musculoskeletal model customized for squatting task. Comput. Methods Biomech. Biomed. Eng..

[26] Lu Y., Mei Q., Peng H.-T., Li J., Wei C., Gu Y. (2020). A Comparative Study on Loadings of the Lower Extremity during Deep Squat in Asian and Caucasian Individuals via OpenSim Musculoskeletal Modelling. BioMed Res. Int..

[27] Butler A.B., Caruntu D.I., Freeman R.A. (2017). Knee Joint Biomechanics for Various Ambulatory Exercises Using Inverse Dynamics in OpenSim. Proceedings of the ASME 2017 International Mechanical Engineering Congress and Exposition. Volume 3: Biomedical and Biotechnology Engineering.

[28] Pizzolato C., Reggiani M., Modenese L., Lloyd D.G. (2016). Real-time inverse kinematics and inverse dynamics for lower limb applications using OpenSim. Comput. Methods Biomech. Biomed. Eng..

[29] Mei Q., Gu Y., Xiang L., Baker J.S., Fernandez J. (2019). Foot Pronation Contributes to Altered Lower Extremity Loading After Long Distance Running. Front. Physiol..

[30] Rajagopal A., Dembia C., DeMers M., Delp D.D., Hicks J.L., Delp S.L. (2016). Full-Body Musculoskeletal Model for Muscle-Driven Simulation of Human Gait. IEEE Trans. Biomed. Eng..

[31] Mei Q., Yaodong G.U., Dong S.U.N., Jianshe L.I., Justin F. (2020). Progress on Biomechanical Research of Image-Based Subject-Specific OpenSim Lower Extremity Musculoskeletal Model. J. Med Biomech..

[32] McKean M.R., Dunn P.K., Burkett B.J. (2010). The Lumbar and Sacrum Movement Pattern during the Back Squat Exercise. J. Strength Cond. Res..

[33] Winter D.A. (2009). Biomechanics and Motor Control of Human Movement.

[34] Delp S.L., Anderson F.C., Arnold A.S., Loan P., Habib A., John C.T., Guendelman E., Thelen D.G. (2007). OpenSim: Open-Source Software to Create and Analyze Dynamic Simulations of Movement. IEEE Trans. Biomed. Eng..

[35] Gallo C., Thompson W., Lewandowski B., Humphreys B., Funk J., Funk N., Weaver A., Perusek G., Sheehan C., Mulugeta L. Computational Modeling Using OpenSim to Simulate a Squat Exercise Motion. Proceedings of the NASA Human Research Program Investigators’ Workshop: Integrated Pathways to Mars.

[36] Golfeshan N., Barnamehei H., Torabigoudarzi M., Karimidastjerdi M., Panahi A., Darman A., Razaghi M., Kharazi M., Jafarloo S.A. (2020). Upper body postures effect on neuromuscular activities of the lower limb during a squat: Musculoskeletal modeling. Gait Posture.

[37] Mei Q., Liangliang X.I.A.N.G., Jianshe L.I., Justin F., Yaodong G.U. (2021). Analysis of Running Ground Reaction Forces Using the One-Dimensional Statistical Parametric Mapping (SPM1d). J. Med Biomech..

[38] Rhea M.R., Kenn J.G., Peterson M.D., Massey D., Simão R., Marin P.J., Favero M., Cardozo D., Krein D. (2016). Joint-Angle Specific Strength Adaptations Influence Improvements in Power in Highly Trained Athletes. Hum. Mov..

[39] Whitting J.W., Meir R.A., Crowley-McHattan Z.J., Holding R.C. (2016). Influence of footwear type on barbell back squat using 50, 70, and 90% of one repetition maximum: A biomechanical analysis. J. Strength Cond. Res..

[40] Swinton P.A., Lloyd R., Keogh J.W., Agouris I., Stewart A.D. (2012). A biomechanical comparison of the traditional squat, powerlifting squat, and box squat. J. Strength Cond. Res..

[41] Lorenz D., Reiman M. (2011). The role and implementation of eccentric training in athletic rehabilitation: Tendinopathy, hamstring strains, and acl reconstruction. Int. J. Sports Phys. Ther..

[42] Markolf K.L., Gorek J.F., Kabo J.M., Shapiro M.S. (1990). Direct measurement of resultant forces in the anterior cruciate ligament. An in vitro study performed with a new experimental technique. J. Bone Jt. Surg. Am. Vol..

[43] Shoepe T., Ramirez D., Rovetti R., Kohler D., Almstedt H. (2011). The Effects of 24 weeks of Resistance Training with Simultaneous Elastic and Free Weight Loading on Muscular Performance of Novice Lifters. J. Hum. Kinet..

[44] Miletello W.M., Beam J.R., Cooper Z.C. (2009). A Biomechanical Analysis of the Squat Between Competitive Collegiate, Competitive High School, and Novice Powerlifters. J. Strength Cond. Res..

[45] Butler R.J., Plisky P.J., Southers C., Scoma C., Kiesel K.B. (2010). Biomechanical analysis of the different classifications of the Functional Movement Screen deep squat test. Sports Biomech..

[46] Chandler T.J., Wilson G.D., Stone M.H. (1989). The effect of the squat exercise on knee stability. Med. Sci. Sports Exerc..

[47] Hasegawa R., Goto F., Watanabe H., Ido H., Okayama N., Islam M.M. (2021). The Relationship between Functional Fitness and Ability to Ride a Bicycle among Community-Dwelling Older Adult in Japan. Phys. Act. Health.

